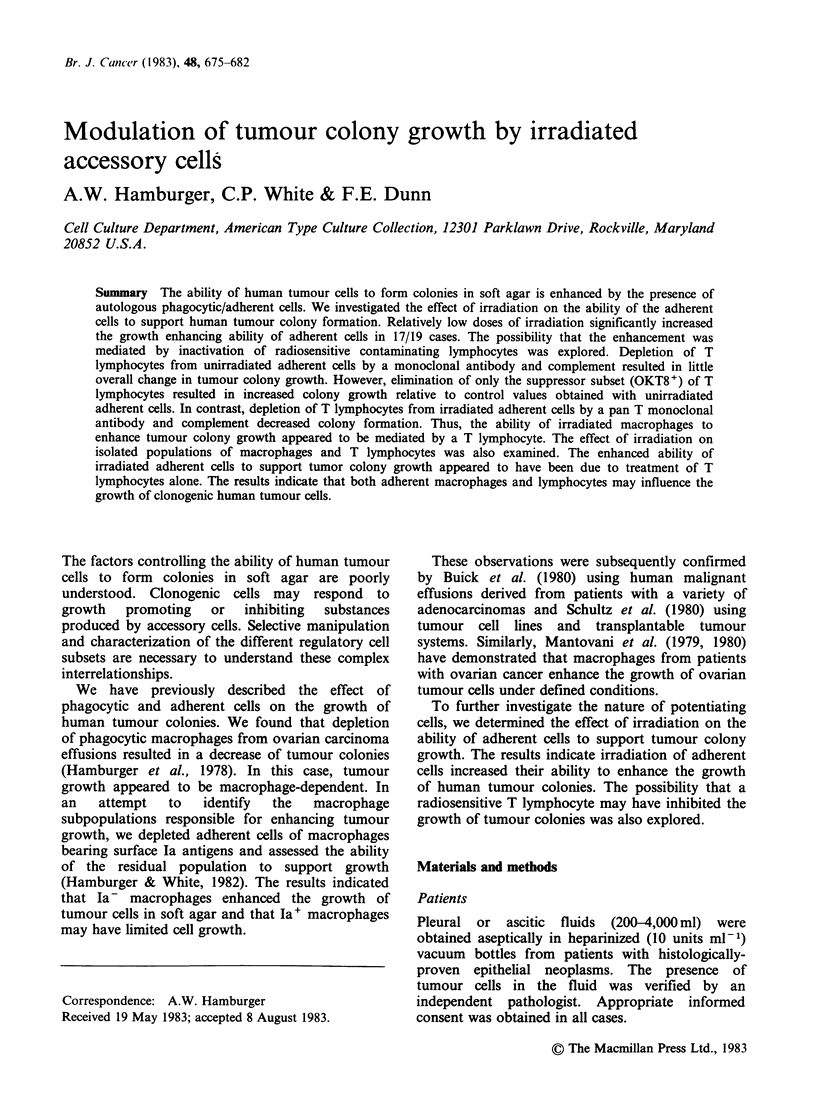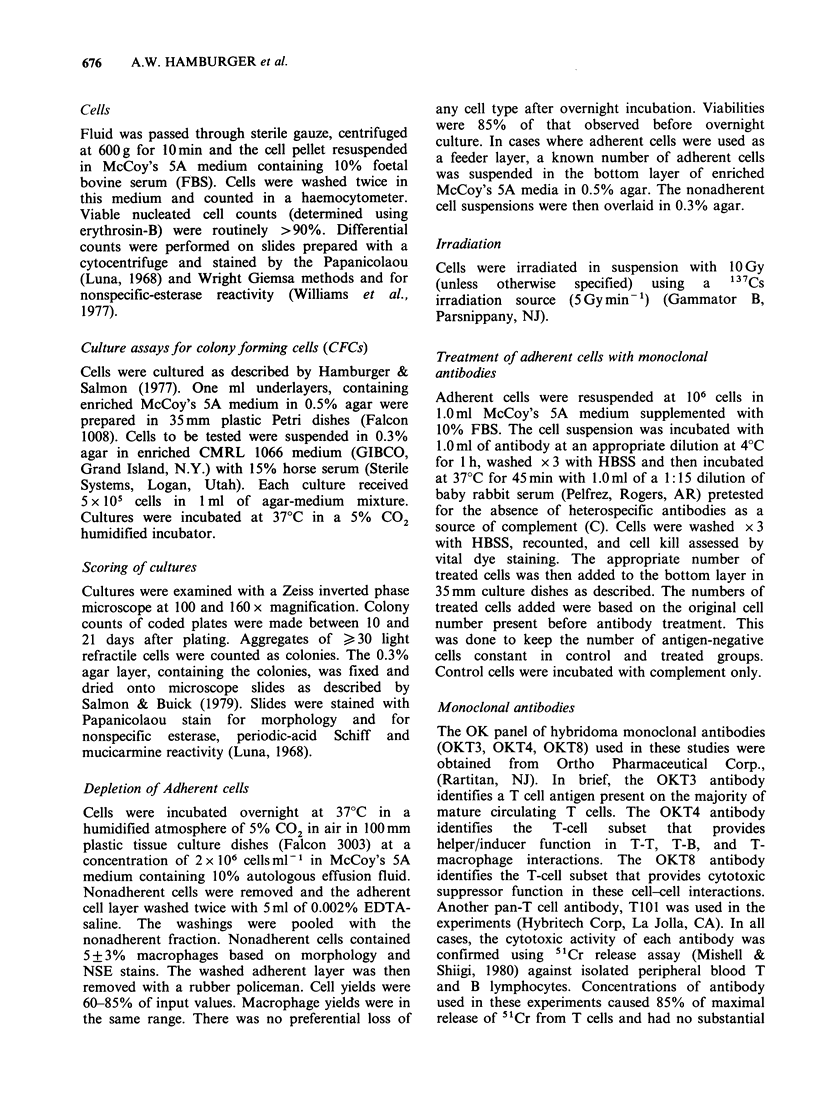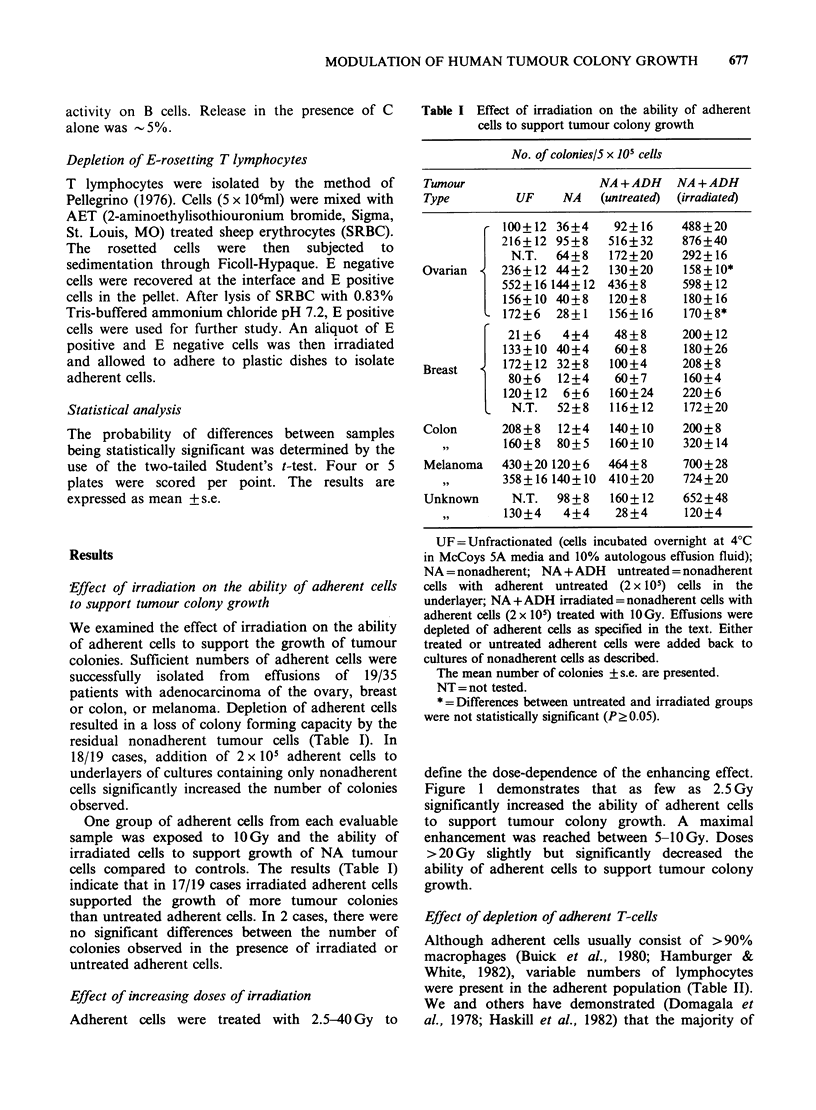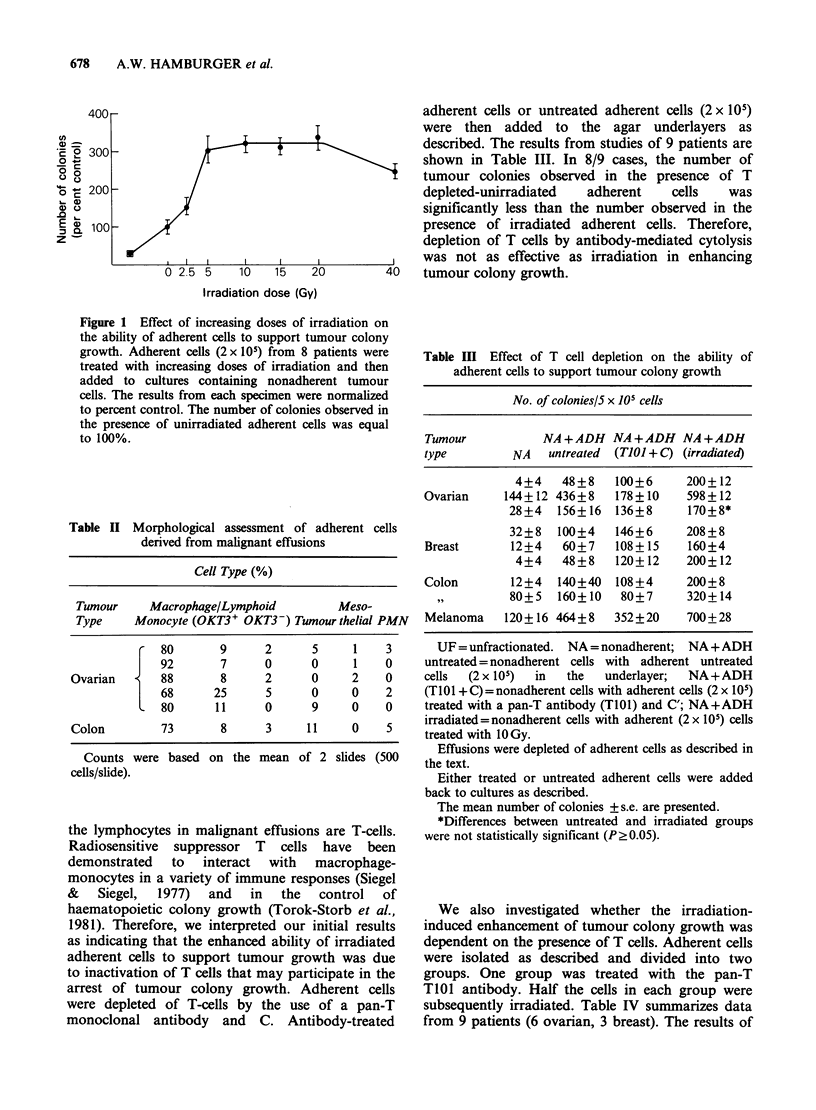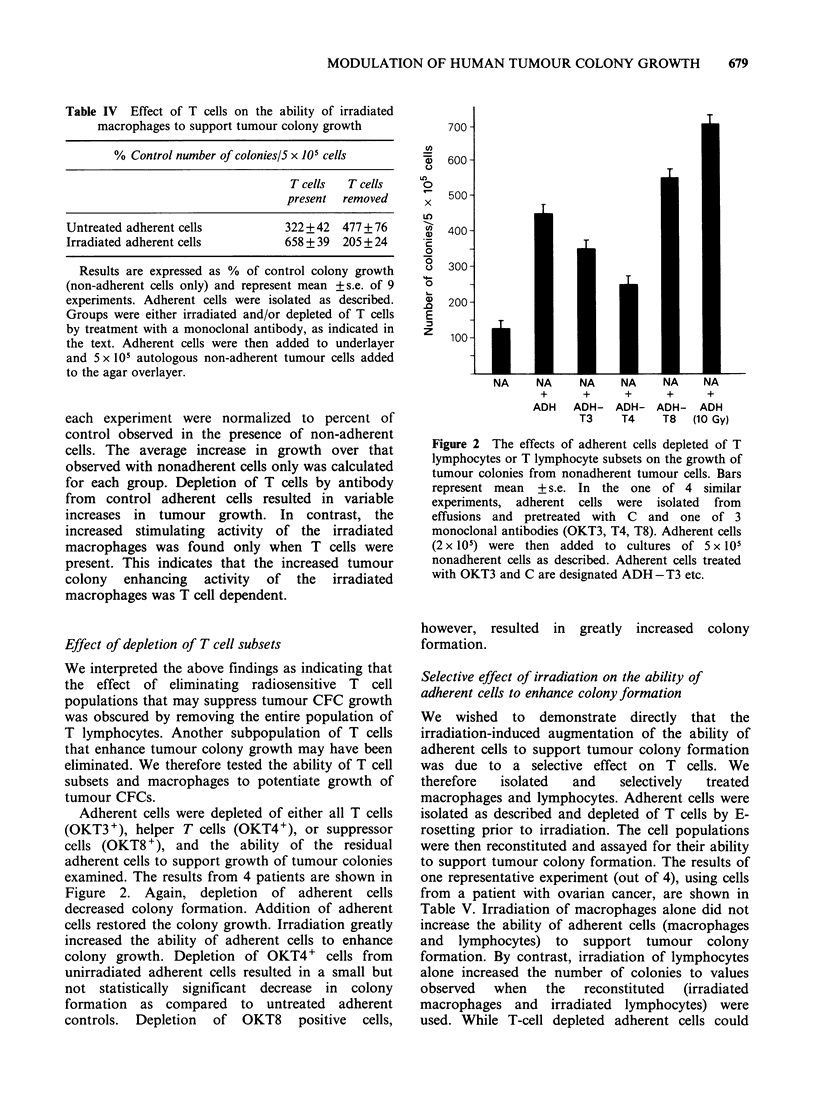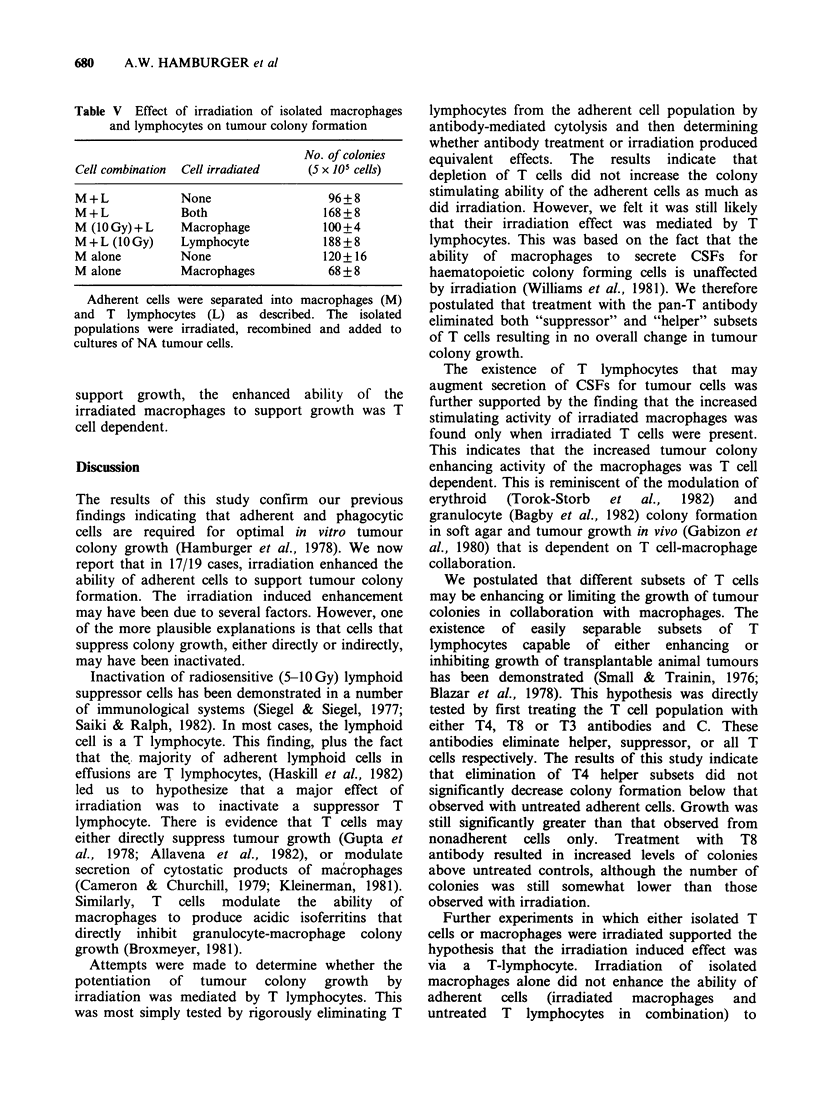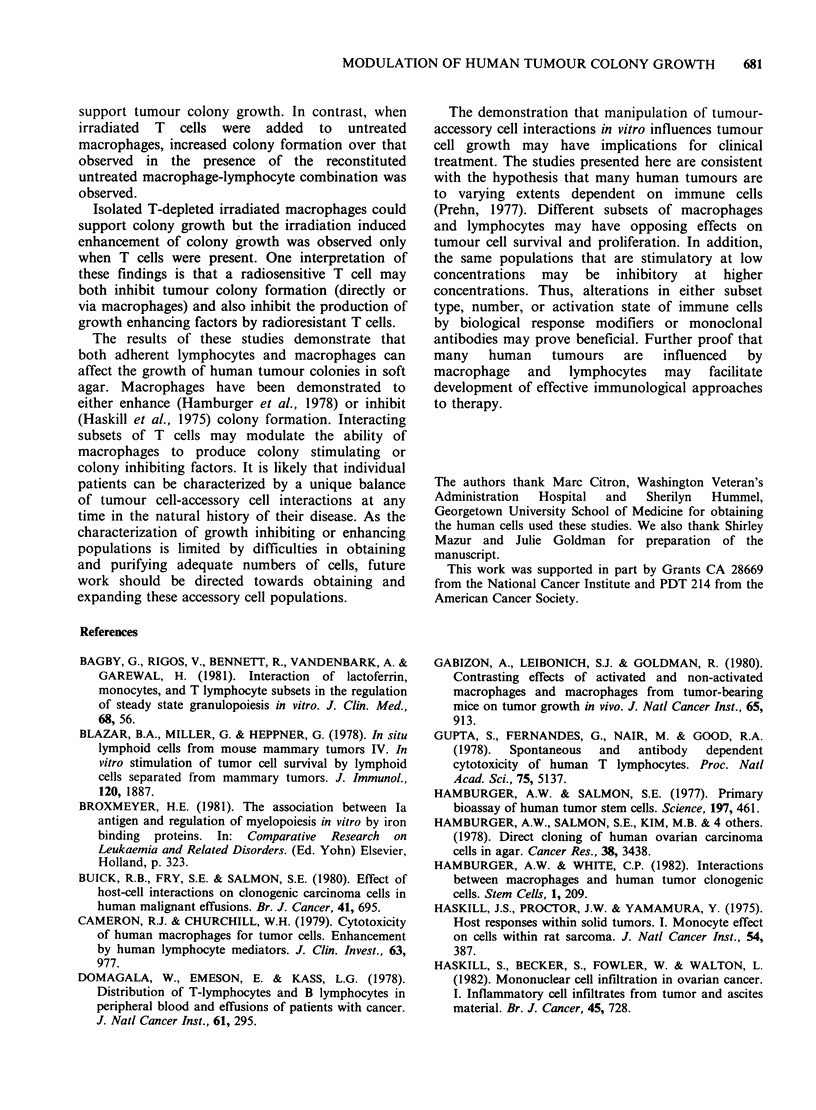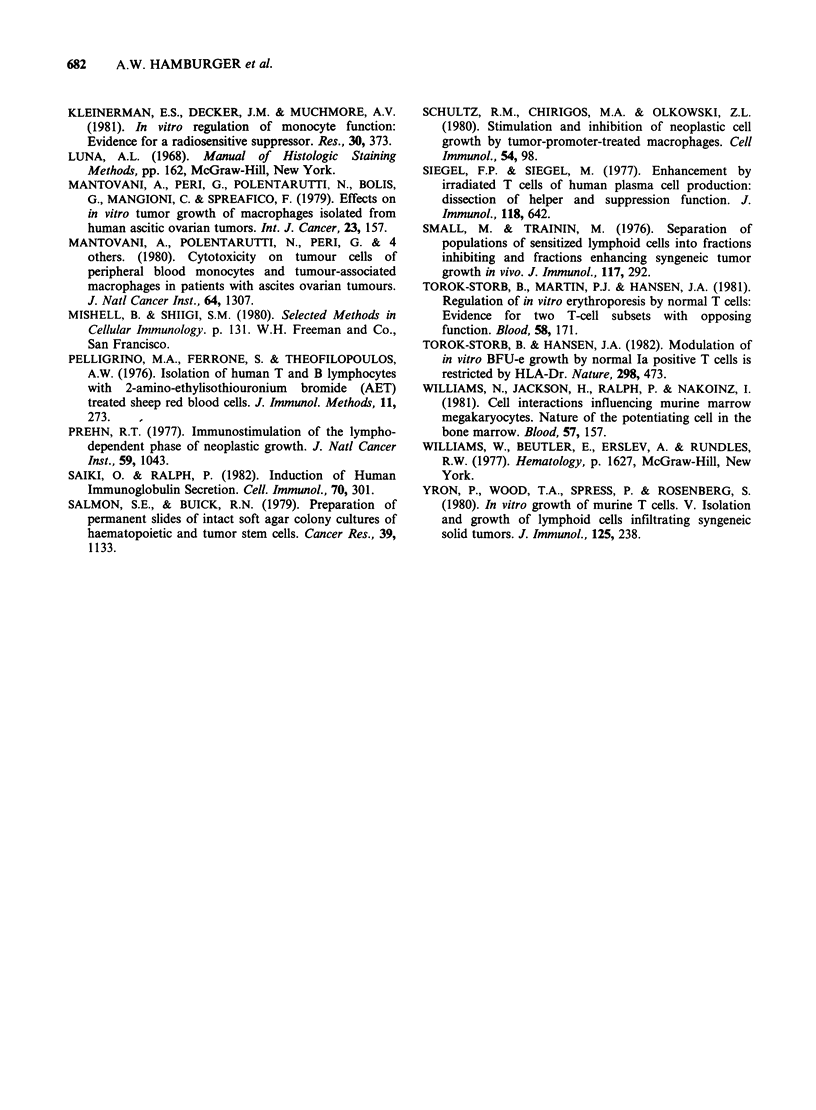# Modulation of tumour colony growth by irradiated accessory cells.

**DOI:** 10.1038/bjc.1983.249

**Published:** 1983-11

**Authors:** A. W. Hamburger, C. P. White, F. E. Dunn

## Abstract

The ability of human tumour cells to form colonies in soft agar is enhanced by the presence of autologous phagocytic/adherent cells. We investigated the effect of irradiation on the ability of the adherent cells to support human tumour colony formation. Relatively low doses of irradiation significantly increased the growth enhancing ability of adherent cells in 17/19 cases. The possibility that the enhancement was mediated by inactivation of radiosensitive contaminating lymphocytes was explored. Depletion of T lymphocytes from unirradiated adherent cells by a monoclonal antibody and complement resulted in little overall change in tumour colony growth. However, elimination of only the suppressor subset (OKT8+) of T lymphocytes resulted in increased colony growth relative to control values obtained with unirradiated adherent cells. In contrast, depletion of T lymphocytes from irradiated adherent cells by a pan T monoclonal antibody and complement decreased colony formation. Thus, the ability of irradiated macrophages to enhance tumour colony growth appeared to be mediated by a T lymphocyte. The effect of irradiation on isolated populations of macrophages and T lymphocytes was also examined. The enhanced ability of irradiated adherent cells to support tumor colony growth appeared to have been due to treatment of T lymphocytes alone. The results indicate that both adherent macrophages and lymphocytes may influence the growth of clonogenic human tumour cells.


Br. J. Cancer (1983), **48**, 675–682

### Modulation of tumour colony growth by irradiated accessory cells

A.W. Hamburger, C.P. White & F.E. Dunn

*Cell Culture Department, American Type Culture Collection, 12301 Parklawn Drive, Rockville, Maryland 20852 U.S.A.*

**Summary** The ability of human tumour cells to form colonies in soft agar is enhanced by the presence of autologous phagocytic/adherent cells. We investigated the effect of irradiation on the ability of the adherent cells to support human tumour colony formation. Relatively low doses of irradiation significantly increased the growth enhancing ability of adherent cells in 17/19 cases. The possibility that the enhancement was mediated by inactivation of radiosensitive contaminating lymphocytes was explored. Depletion of T lymphocytes from unirradiated adherent cells by a monoclonal antibody and complement resulted in little overall change in tumour colony growth. However, elimination of only the suppressor subset (OKT8$^+$) of T lymphocytes resulted in increased colony growth relative to control values obtained with unirradiated adherent cells. In contrast, depletion of T lymphocytes from irradiated adherent cells by a pan T monoclonal antibody and complement decreased colony formation. Thus, the ability of irradiated macrophages to enhance tumour colony growth appeared to be mediated by a T lymphocyte. The effect of irradiation on isolated populations of macrophages and T lymphocytes was also examined. The enhanced ability of irradiated adherent cells to support tumor colony growth appeared to have been due to treatment of T lymphocytes alone. The results indicate that both adherent macrophages and lymphocytes may influence the growth of clonogenic human tumour cells.

The factors controlling the ability of human tumour cells to form colonies in soft agar are poorly understood. Clonogenic cells may respond to growth promoting or inhibiting substances produced by accessory cells. Selective manipulation and characterization of the different regulatory cell subsets are necessary to understand these complex interrelationships.

We have previously described the effect of phagocytic and adherent cells on the growth of human tumour colonies. We found that depletion of phagocytic macrophages from ovarian carcinoma effusions resulted in a decrease of tumour colonies (Hamburger *et al.,* 1978). In this case, tumour growth appeared to be macrophage-dependent. In an attempt to identify the macrophage subpopulations responsible for enhancing tumour growth, we depleted adherent cells of macrophages bearing surface Ia antigens and assessed the ability of the residual population to support growth (Hamburger & White, 1982). The results indicated that Ia$^-$ macrophages enhanced the growth of tumour cells in soft agar and that Ia$^+$ macrophages may have limited cell growth.

Correspondence: A.W. Hamburger
Received 19 May 1983; accepted 8 August 1983.

These observations were subsequently confirmed by Buick *et al.* (1980) using human malignant effusions derived from patients with a variety of adenocarcinomas and Schultz *et al.* (1980) using tumour cell lines and transplantable tumour systems. Similarly, Mantovani *et al.* (1979, 1980) have demonstrated that macrophages from patients with ovarian cancer enhance the growth of ovarian tumour cells under defined conditions.

To further investigate the nature of potentiating cells, we determined the effect of irradiation on the ability of adherent cells to support tumour colony growth. The results indicate irradiation of adherent cells increased their ability to enhance the growth of human tumour colonies. The possibility that a radiosensitive T lymphocyte may have inhibited the growth of tumour colonies was also explored.

### Materials and methods

### *Patients*

Pleural or ascitic fluids (200–4,000 ml) were obtained aseptically in heparinized (10 units ml$^{-1}$) vacuum bottles from patients with histologically-proven epithelial neoplasms. The presence of tumour cells in the fluid was verified by an independent pathologist. Appropriate informed consent was obtained in all cases.

© The Macmillan Press Ltd., 1983

*Cells*

Fluid was passed through sterile gauze, centrifuged at 600 g for 10 min and the cell pellet resuspended in McCoy's 5A medium containing 10% foetal bovine serum (FBS). Cells were washed twice in this medium and counted in a haemocytometer. Viable nucleated cell counts (determined using erythrosin-B) were routinely >90%. Differential counts were performed on slides prepared with a cytocentrifuge and stained by the Papanicolaou (Luna, 1968) and Wright Giemsa methods and for nonspecific-esterase reactivity (Williams *et al.*, 1977).

*Culture assays for colony forming cells (CFCs)*

Cells were cultured as described by Hamburger & Salmon (1977). One ml underlayers, containing enriched McCoy's 5A medium in 0.5% agar were prepared in 35 mm plastic Petri dishes (Falcon 1008). Cells to be tested were suspended in 0.3% agar in enriched CMRL 1066 medium (GIBCO, Grand Island, N.Y.) with 15% horse serum (Sterile Systems, Logan, Utah). Each culture received $5 \times 10^5$ cells in 1 ml of agar-medium mixture. Cultures were incubated at 37°C in a 5% $CO_2$ humidified incubator.

*Scoring of cultures*

Cultures were examined with a Zeiss inverted phase microscope at 100 and 160 × magnification. Colony counts of coded plates were made between 10 and 21 days after plating. Aggregates of ⩾30 light refractile cells were counted as colonies. The 0.3% agar layer, containing the colonies, was fixed and dried onto microscope slides as described by Salmon & Buick (1979). Slides were stained with Papanicolaou stain for morphology and for nonspecific esterase, periodic-acid Schiff and mucicarmine reactivity (Luna, 1968).

*Depletion of Adherent cells*

Cells were incubated overnight at 37°C in a humidified atmosphere of 5% $CO_2$ in air in 100 mm plastic tissue culture dishes (Falcon 3003) at a concentration of $2 \times 10^6$ cells $ml^{-1}$ in McCoy's 5A medium containing 10% autologous effusion fluid. Nonadherent cells were removed and the adherent cell layer washed twice with 5 ml of 0.002% EDTA-saline. The washings were pooled with the nonadherent fraction. Nonadherent cells contained $5 \pm 3$% macrophages based on morphology and NSE stains. The washed adherent layer was then removed with a rubber policeman. Cell yields were 60–85% of input values. Macrophage yields were in the same range. There was no preferential loss of any cell type after overnight incubation. Viabilities were 85% of that observed before overnight culture. In cases where adherent cells were used as a feeder layer, a known number of adherent cells was suspended in the bottom layer of enriched McCoy's 5A media in 0.5% agar. The nonadherent cell suspensions were then overlaid in 0.3% agar.

*Irradiation*

Cells were irradiated in suspension with 10 Gy (unless otherwise specified) using a $^{137}Cs$ irradiation source (5 Gy $min^{-1}$) (Gammator B, Parsnippany, NJ).

*Treatment of adherent cells with monoclonal antibodies*

Adherent cells were resuspended at $10^6$ cells in 1.0 ml McCoy's 5A medium supplemented with 10% FBS. The cell suspension was incubated with 1.0 ml of antibody at an appropriate dilution at 4°C for 1 h, washed × 3 with HBSS and then incubated at 37°C for 45 min with 1.0 ml of a 1:15 dilution of baby rabbit serum (Pelfrez, Rogers, AR) pretested for the absence of heterospecific antibodies as a source of complement (C). Cells were washed × 3 with HBSS, recounted, and cell kill assessed by vital dye staining. The appropriate number of treated cells was then added to the bottom layer in 35 mm culture dishes as described. The numbers of treated cells added were based on the original cell number present before antibody treatment. This was done to keep the number of antigen-negative cells constant in control and treated groups. Control cells were incubated with complement only.

*Monoclonal antibodies*

The OK panel of hybridoma monoclonal antibodies (OKT3, OKT4, OKT8) used in these studies were obtained from Ortho Pharmaceutical Corp., (Rartitan, NJ). In brief, the OKT3 antibody identifies a T cell antigen present on the majority of mature circulating T cells. The OKT4 antibody identifies the T-cell subset that provides helper/inducer function in T-T, T-B, and T-macrophage interactions. The OKT8 antibody identifies the T-cell subset that provides cytotoxic suppressor function in these cell–cell interactions. Another pan-T cell antibody, T101 was used in the experiments (Hybritech Corp, La Jolla, CA). In all cases, the cytotoxic activity of each antibody was confirmed using $^{51}Cr$ release assay (Mishell & Shiigi, 1980) against isolated peripheral blood T and B lymphocytes. Concentrations of antibody used in these experiments caused 85% of maximal release of $^{51}Cr$ from T cells and had no substantial activity on B cells. Release in the presence of C alone was ~5%.

*Depletion of E-rosetting T lymphocytes*

T lymphocytes were isolated by the method of Pellegrino (1976). Cells ($5 \times 10^6$ml) were mixed with AET (2-aminoethylisothiouronium bromide, Sigma, St. Louis, MO) treated sheep erythrocytes (SRBC). The rosetted cells were then subjected to sedimentation through Ficoll-Hypaque. E negative cells were recovered at the interface and E positive cells in the pellet. After lysis of SRBC with 0.83% Tris-buffered ammonium chloride pH 7.2, E positive cells were used for further study. An aliquot of E positive and E negative cells was then irradiated and allowed to adhere to plastic dishes to isolate adherent cells.

*Statistical analysis*

The probability of differences between samples being statistically significant was determined by the use of the two-tailed Student's *t*-test. Four or 5 plates were scored per point. The results are expressed as mean ±s.e.

### Results

*Effect of irradiation on the ability of adherent cells to support tumour colony growth*

We examined the effect of irradiation on the ability of adherent cells to support the growth of tumour colonies. Sufficient numbers of adherent cells were successfully isolated from effusions of 19/35 patients with adenocarcinoma of the ovary, breast or colon, or melanoma. Depletion of adherent cells resulted in a loss of colony forming capacity by the residual nonadherent tumour cells (Table I). In 18/19 cases, addition of $2 \times 10^5$ adherent cells to underlayers of cultures containing only nonadherent cells significantly increased the number of colonies observed.

One group of adherent cells from each evaluable sample was exposed to 10 Gy and the ability of irradiated cells to support growth of NA tumour cells compared to controls. The results (Table I) indicate that in 17/19 cases irradiated adherent cells supported the growth of more tumour colonies than untreated adherent cells. In 2 cases, there were no significant differences between the number of colonies observed in the presence of irradiated or untreated adherent cells.

*Effect of increasing doses of irradiation*

Adherent cells were treated with 2.5–40 Gy to define the dose-dependence of the enhancing effect. Figure 1 demonstrates that as few as 2.5 Gy significantly increased the ability of adherent cells to support tumour colony growth. A maximal enhancement was reached between 5–10 Gy. Doses >20 Gy slightly but significantly decreased the ability of adherent cells to support tumour colony growth.

**Table I** Effect of irradiation on the ability of adherent cells to support tumour colony growth

| | *No. of colonies/5 × 10⁵ cells* | | | |
|---|---|---|---|---|
| *Tumour Type* | *UF* | *NA* | *NA + ADH (untreated)* | *NA + ADH (irradiated)* |
| Ovarian | 100±12 | 36±4 | 92±16 | 488±20 |
| | 216±12 | 95±8 | 516±32 | 876±40 |
| | N.T. | 64±8 | 172±20 | 292±16 |
| | 236±12 | 44±2 | 130±20 | 158±10* |
| | 552±16 | 144±12 | 436±8 | 598±12 |
| | 156±10 | 40±8 | 120±8 | 180±16 |
| | 172±6 | 28±1 | 156±16 | 170±8* |
| Breast | 21±6 | 4±4 | 48±8 | 200±12 |
| | 133±10 | 40±4 | 60±8 | 180±26 |
| | 172±12 | 32±8 | 100±4 | 208±8 |
| | 80±6 | 12±4 | 60±7 | 160±4 |
| | 120±12 | 6±6 | 160±24 | 220±6 |
| | N.T. | 52±8 | 116±12 | 172±20 |
| Colon | 208±8 | 12±4 | 140±10 | 200±8 |
| " | 160±8 | 80±5 | 160±10 | 320±14 |
| Melanoma | 430±20 | 120±6 | 464±8 | 700±28 |
| " | 358±16 | 140±10 | 410±20 | 724±20 |
| Unknown | N.T. | 98±8 | 160±12 | 652±48 |
| " | 130±4 | 4±4 | 28±4 | 120±4 |

UF = Unfractionated (cells incubated overnight at 4°C in McCoys 5A media and 10% autologous effusion fluid); NA = nonadherent; NA + ADH untreated = nonadherent cells with adherent untreated ($2 \times 10^5$) cells in the underlayer; NA + ADH irradiated = nonadherent cells with adherent cells ($2 \times 10^5$) treated with 10 Gy. Effusions were depleted of adherent cells as specified in the text. Either treated or untreated adherent cells were added back to cultures of nonadherent cells as described.

The mean number of colonies ±s.e. are presented.

NT = not tested.

* = Differences between untreated and irradiated groups were not statistically significant ($P \geq 0.05$).

*Effect of depletion of adherent T-cells*

Although adherent cells usually consist of >90% macrophages (Buick *et al.*, 1980; Hamburger & White, 1982), variable numbers of lymphocytes were present in the adherent population (Table II). We and others have demonstrated (Domagala *et al.*, 1978; Haskill *et al.*, 1982) that the majority of

**Figure 1** Effect of increasing doses of irradiation on the ability of adherent cells to support tumour colony growth. Adherent cells ($2 \times 10^5$) from 8 patients were treated with increasing doses of irradiation and then added to cultures containing nonadherent tumour cells. The results from each specimen were normalized to percent control. The number of colonies observed in the presence of unirradiated adherent cells was equal to 100%.

**Table II** Morphological assessment of adherent cells derived from malignant effusions

| Tumour Type | Cell Type (%) Macrophage/Monocyte | Lymphoid ($OKT3^+$ | $OKT3^-$) | Tumour | Mesothelial | PMN |
|---|---|---|---|---|---|---|
| Ovarian | 80 | 9 | 2 | 5 | 1 | 3 |
| | 92 | 7 | 0 | 0 | 1 | 0 |
| | 88 | 8 | 2 | 0 | 2 | 0 |
| | 68 | 25 | 5 | 0 | 0 | 2 |
| | 80 | 11 | 0 | 9 | 0 | 0 |
| Colon | 73 | 8 | 3 | 11 | 0 | 5 |

Counts were based on the mean of 2 slides (500 cells/slide).

the lymphocytes in malignant effusions are T-cells. Radiosensitive suppressor T cells have been demonstrated to interact with macrophage-monocytes in a variety of immune responses (Siegel & Siegel, 1977) and in the control of haematopoietic colony growth (Torok-Storb et al., 1981). Therefore, we interpreted our initial results as indicating that the enhanced ability of irradiated adherent cells to support tumour growth was due to inactivation of T cells that may participate in the arrest of tumour colony growth. Adherent cells were depleted of T-cells by the use of a pan-T monoclonal antibody and C. Antibody-treated adherent cells or untreated adherent cells ($2 \times 10^5$) were then added to the agar underlayers as described. The results from studies of 9 patients are shown in Table III. In 8/9 cases, the number of tumour colonies observed in the presence of T depleted-unirradiated adherent cells was significantly less than the number observed in the presence of irradiated adherent cells. Therefore, depletion of T cells by antibody-mediated cytolysis was not as effective as irradiation in enhancing tumour colony growth.

**Table III** Effect of T cell depletion on the ability of adherent cells to support tumour colony growth

| Tumour type | No. of colonies/$5 \times 10^5$ cells NA | NA+ADH untreated | NA+ADH (T101+C) | NA+ADH (irradiated) |
|---|---|---|---|---|
| | 4±4 | 48±8 | 100±6 | 200±12 |
| Ovarian | 144±12 | 436±8 | 178±10 | 598±12 |
| | 28±4 | 156±16 | 136±8 | 170±8* |
| | 32±8 | 100±4 | 146±6 | 208±8 |
| Breast | 12±4 | 60±7 | 108±15 | 160±4 |
| | 4±4 | 48±8 | 120±12 | 200±12 |
| Colon | 12±4 | 140±40 | 108±4 | 200±8 |
| ” | 80±5 | 160±10 | 80±7 | 320±14 |
| Melanoma | 120±16 | 464±8 | 352±20 | 700±28 |

UF = unfractionated. NA = nonadherent; NA+ADH untreated = nonadherent cells with adherent untreated cells ($2 \times 10^5$) in the underlayer; NA+ADH (T101+C) = nonadherent cells with adherent cells ($2 \times 10^5$) treated with a pan-T antibody (T101) and C′; NA+ADH irradiated = nonadherent cells with adherent ($2 \times 10^5$) cells treated with 10 Gy.

Effusions were depleted of adherent cells as described in the text.

Either treated or untreated adherent cells were added back to cultures as described.

The mean number of colonies ±s.e. are presented.

*Differences between untreated and irradiated groups were not statistically significant ($P \geq 0.05$).

We also investigated whether the irradiation-induced enhancement of tumour colony growth was dependent on the presence of T cells. Adherent cells were isolated as described and divided into two groups. One group was treated with the pan-T T101 antibody. Half the cells in each group were subsequently irradiated. Table IV summarizes data from 9 patients (6 ovarian, 3 breast). The results of

**Table IV** Effect of T cells on the ability of irradiated macrophages to support tumour colony growth

| | *% Control number of colonies/5 × 10⁵ cells* | |
|---|---|---|
| | *T cells present* | *T cells removed* |
| Untreated adherent cells | 322±42 | 477±76 |
| Irradiated adherent cells | 658±39 | 205±24 |

Results are expressed as % of control colony growth (non-adherent cells only) and represent mean ±s.e. of 9 experiments. Adherent cells were isolated as described. Groups were either irradiated and/or depleted of T cells by treatment with a monoclonal antibody, as indicated in the text. Adherent cells were then added to underlayer and $5 \times 10^5$ autologous non-adherent tumour cells added to the agar overlayer.

**Figure 2** The effects of adherent cells depleted of T lymphocytes or T lymphocyte subsets on the growth of tumour colonies from nonadherent tumour cells. Bars represent mean ±s.e. In the one of 4 similar experiments, adherent cells were isolated from effusions and pretreated with C and one of 3 monoclonal antibodies (OKT3, T4, T8). Adherent cells $(2 \times 10^5)$ were then added to cultures of $5 \times 10^5$ nonadherent cells as described. Adherent cells treated with OKT3 and C are designated ADH−T3 etc.

each experiment were normalized to percent of control observed in the presence of non-adherent cells. The average increase in growth over that observed with nonadherent cells only was calculated for each group. Depletion of T cells by antibody from control adherent cells resulted in variable increases in tumour growth. In contrast, the increased stimulating activity of the irradiated macrophages was found only when T cells were present. This indicates that the increased tumour colony enhancing activity of the irradiated macrophages was T cell dependent.

### *Effect of depletion of T cell subsets*

We interpreted the above findings as indicating that the effect of eliminating radiosensitive T cell populations that may suppress tumour CFC growth was obscured by removing the entire population of T lymphocytes. Another subpopulation of T cells that enhance tumour colony growth may have been eliminated. We therefore tested the ability of T cell subsets and macrophages to potentiate growth of tumour CFCs.

Adherent cells were depleted of either all T cells ($OKT3^+$), helper *T* cells ($OKT4^+$), or suppressor cells ($OKT8^+$), and the ability of the residual adherent cells to support growth of tumour colonies examined. The results from 4 patients are shown in Figure 2. Again, depletion of adherent cells decreased colony formation. Addition of adherent cells restored the colony growth. Irradiation greatly increased the ability of adherent cells to enhance colony growth. Depletion of $OKT4^+$ cells from unirradiated adherent cells resulted in a small but not statistically significant decrease in colony formation as compared to untreated adherent controls. Depletion of OKT8 positive cells, however, resulted in greatly increased colony formation.

### *Selective effect of irradiation on the ability of adherent cells to enhance colony formation*

We wished to demonstrate directly that the irradiation-induced augmentation of the ability of adherent cells to support tumour colony formation was due to a selective effect on T cells. We therefore isolated and selectively treated macrophages and lymphocytes. Adherent cells were isolated as described and depleted of T cells by E-rosetting prior to irradiation. The cell populations were then reconstituted and assayed for their ability to support tumour colony formation. The results of one representative experiment (out of 4), using cells from a patient with ovarian cancer, are shown in Table V. Irradiation of macrophages alone did not increase the ability of adherent cells (macrophages and lymphocytes) to support tumour colony formation. By contrast, irradiation of lymphocytes alone increased the number of colonies to values observed when the reconstituted (irradiated macrophages and irradiated lymphocytes) were used. While T-cell depleted adherent cells could

**Table V** Effect of irradiation of isolated macrophages and lymphocytes on tumour colony formation

| *Cell combination* | *Cell irradiated* | *No. of colonies ($5\times10^5$ cells)* |
|---|---|---|
| M+L | None | $96\pm8$ |
| M+L | Both | $168\pm8$ |
| M (10 Gy)+L | Macrophage | $100\pm4$ |
| M+L (10 Gy) | Lymphocyte | $188\pm8$ |
| M alone | None | $120\pm16$ |
| M alone | Macrophages | $68\pm8$ |

Adherent cells were separated into macrophages (M) and T lymphocytes (L) as described. The isolated populations were irradiated, recombined and added to cultures of NA tumour cells.

support growth, the enhanced ability of the irradiated macrophages to support growth was T cell dependent.

### Discussion

The results of this study confirm our previous findings indicating that adherent and phagocytic cells are required for optimal *in vitro* tumour colony growth (Hamburger *et al.*, 1978). We now report that in 17/19 cases, irradiation enhanced the ability of adherent cells to support tumour colony formation. The irradiation induced enhancement may have been due to several factors. However, one of the more plausible explanations is that cells that suppress colony growth, either directly or indirectly, may have been inactivated.

Inactivation of radiosensitive (5–10 Gy) lymphoid suppressor cells has been demonstrated in a number of immunological systems (Siegel & Siegel, 1977; Saiki & Ralph, 1982). In most cases, the lymphoid cell is a T lymphocyte. This finding, plus the fact that the majority of adherent lymphoid cells in effusions are T lymphocytes, (Haskill *et al.*, 1982) led us to hypothesize that a major effect of irradiation was to inactivate a suppressor T lymphocyte. There is evidence that T cells may either directly suppress tumour growth (Gupta *et al.*, 1978; Allavena *et al.*, 1982), or modulate secretion of cytostatic products of macrophages (Cameron & Churchill, 1979; Kleinerman, 1981). Similarly, T cells modulate the ability of macrophages to produce acidic isoferritins that directly inhibit granulocyte-macrophage colony growth (Broxmeyer, 1981).

Attempts were made to determine whether the potentiation of tumour colony growth by irradiation was mediated by T lymphocytes. This was most simply tested by rigorously eliminating T lymphocytes from the adherent cell population by antibody-mediated cytolysis and then determining whether antibody treatment or irradiation produced equivalent effects. The results indicate that depletion of T cells did not increase the colony stimulating ability of the adherent cells as much as did irradiation. However, we felt it was still likely that their irradiation effect was mediated by T lymphocytes. This was based on the fact that the ability of macrophages to secrete CSFs for haematopoietic colony forming cells is unaffected by irradiation (Williams *et al.*, 1981). We therefore postulated that treatment with the pan-T antibody eliminated both "suppressor" and "helper" subsets of T cells resulting in no overall change in tumour colony growth.

The existence of T lymphocytes that may augment secretion of CSFs for tumour cells was further supported by the finding that the increased stimulating activity of irradiated macrophages was found only when irradiated T cells were present. This indicates that the increased tumour colony enhancing activity of the macrophages was T cell dependent. This is reminiscent of the modulation of erythroid (Torok-Storb *et al.*, 1982) and granulocyte (Bagby *et al.*, 1982) colony formation in soft agar and tumour growth *in vivo* (Gabizon *et al.*, 1980) that is dependent on T cell-macrophage collaboration.

We postulated that different subsets of T cells may be enhancing or limiting the growth of tumour colonies in collaboration with macrophages. The existence of easily separable subsets of T lymphocytes capable of either enhancing or inhibiting growth of transplantable animal tumours has been demonstrated (Small & Trainin, 1976; Blazar *et al.*, 1978). This hypothesis was directly tested by first treating the T cell population with either T4, T8 or T3 antibodies and C. These antibodies eliminate helper, suppressor, or all T cells respectively. The results of this study indicate that elimination of T4 helper subsets did not significantly decrease colony formation below that observed with untreated adherent cells. Growth was still significantly greater than that observed from nonadherent cells only. Treatment with T8 antibody resulted in increased levels of colonies above untreated controls, although the number of colonies was still somewhat lower than those observed with irradiation.

Further experiments in which either isolated T cells or macrophages were irradiated supported the hypothesis that the irradiation induced effect was via a T-lymphocyte. Irradiation of isolated macrophages alone did not enhance the ability of adherent cells (irradiated macrophages and untreated T lymphocytes in combination) to support tumour colony growth. In contrast, when irradiated T cells were added to untreated macrophages, increased colony formation over that observed in the presence of the reconstituted untreated macrophage-lymphocyte combination was observed.

Isolated T-depleted irradiated macrophages could support colony growth but the irradiation induced enhancement of colony growth was observed only when T cells were present. One interpretation of these findings is that a radiosensitive T cell may both inhibit tumour colony formation (directly or via macrophages) and also inhibit the production of growth enhancing factors by radioresistant T cells.

The results of these studies demonstrate that both adherent lymphocytes and macrophages can affect the growth of human tumour colonies in soft agar. Macrophages have been demonstrated to either enhance (Hamburger *et al.*, 1978) or inhibit (Haskill *et al.*, 1975) colony formation. Interacting subsets of T cells may modulate the ability of macrophages to produce colony stimulating or colony inhibiting factors. It is likely that individual patients can be characterized by a unique balance of tumour cell-accessory cell interactions at any time in the natural history of their disease. As the characterization of growth inhibiting or enhancing populations is limited by difficulties in obtaining and purifying adequate numbers of cells, future work should be directed towards obtaining and expanding these accessory cell populations.

The demonstration that manipulation of tumour-accessory cell interactions *in vitro* influences tumour cell growth may have implications for clinical treatment. The studies presented here are consistent with the hypothesis that many human tumours are to varying extents dependent on immune cells (Prehn, 1977). Different subsets of macrophages and lymphocytes may have opposing effects on tumour cell survival and proliferation. In addition, the same populations that are stimulatory at low concentrations may be inhibitory at higher concentrations. Thus, alterations in either subset type, number, or activation state of immune cells by biological response modifiers or monoclonal antibodies may prove beneficial. Further proof that many human tumours are influenced by macrophage and lymphocytes may facilitate development of effective immunological approaches to therapy.

The authors thank Marc Citron, Washington Veteran's Administration Hospital and Sherilyn Hummel, Georgetown University School of Medicine for obtaining the human cells used these studies. We also thank Shirley Mazur and Julie Goldman for preparation of the manuscript.

This work was supported in part by Grants CA 28669 from the National Cancer Institute and PDT 214 from the American Cancer Society.

**References**

BAGBY, G., RIGOS, V., BENNETT, R., VANDENBARK, A. & GAREWAL, H. (1981). Interaction of lactoferrin, monocytes, and T lymphocyte subsets in the regulation of steady state granulopoiesis *in vitro*. *J. Clin. Med.*, **68**, 56.

BLAZAR, B.A., MILLER, G. & HEPPNER, G. (1978). *In situ* lymphoid cells from mouse mammary tumors IV. *In vitro* stimulation of tumor cell survival by lymphoid cells separated from mammary tumors. *J. Immunol.*, **120**, 1887.

BROXMEYER, H.E. (1981). The association between Ia antigen and regulation of myelopoiesis *in vitro* by iron binding proteins. In: *Comparative Research on Leukaemia and Related Disorders*. (Ed. Yohn) Elsevier, Holland, p. 323.

BUICK, R.B., FRY, S.E. & SALMON, S.E. (1980). Effect of host-cell interactions on clonogenic carcinoma cells in human malignant effusions. *Br. J. Cancer*, **41**, 695.

CAMERON, R.J. & CHURCHILL, W.H. (1979). Cytotoxicity of human macrophages for tumor cells. Enhancement by human lymphocyte mediators. *J. Clin. Invest.*, **63**, 977.

DOMAGALA, W., EMESON, E. & KASS, L.G. (1978). Distribution of T-lymphocytes and B lymphocytes in peripheral blood and effusions of patients with cancer. *J. Natl Cancer Inst.*, **61**, 295.

GABIZON, A., LEIBONICH, S.J. & GOLDMAN, R. (1980). Contrasting effects of activated and non-activated macrophages and macrophages from tumor-bearing mice on tumor growth *in vivo*. *J. Natl Cancer Inst.*, **65**, 913.

GUPTA, S., FERNANDES, G., NAIR, M. & GOOD, R.A. (1978). Spontaneous and antibody dependent cytotoxicity of human T lymphocytes. *Proc. Natl Acad. Sci.*, **75**, 5137.

HAMBURGER, A.W. & SALMON, S.E. (1977). Primary bioassay of human tumor stem cells. *Science*, **197**, 461.

HAMBURGER, A.W., SALMON, S.E., KIM, M.B. & 4 others. (1978). Direct cloning of human ovarian carcinoma cells in agar. *Cancer Res.*, **38**, 3438.

HAMBURGER, A.W. & WHITE, C.P. (1982). Interactions between macrophages and human tumor clonogenic cells. *Stem Cells*, **1**, 209.

HASKILL, J.S., PROCTOR, J.W. & YAMAMURA, Y. (1975). Host responses within solid tumors. I. Monocyte effect on cells within rat sarcoma. *J. Natl Cancer Inst.*, **54**, 387.

HASKILL, S., BECKER, S., FOWLER, W. & WALTON, L. (1982). Mononuclear cell infiltration in ovarian cancer. I. Inflammatory cell infiltrates from tumor and ascites material. *Br. J. Cancer*, **45**, 728.

KLEINERMAN, E.S., DECKER, J.M. & MUCHMORE, A.V. (1981). *In vitro* regulation of monocyte function: Evidence for a radiosensitive suppressor. *Res.*, **30**, 373.

LUNA, A.L. (1968). *Manual of Histologic Staining Methods,* pp. 162, McGraw-Hill, New York.

MANTOVANI, A., PERI, G., POLENTARUTTI, N., BOLIS, G., MANGIONI, C. & SPREAFICO, F. (1979). Effects on *in vitro* tumor growth of macrophages isolated from human ascitic ovarian tumors. *Int. J. Cancer,* **23,** 157.

MANTOVANI, A., POLENTARUTTI, N., PERI, G. & 4 others. (1980). Cytotoxicity on tumour cells of peripheral blood monocytes and tumour-associated macrophages in patients with ascites ovarian tumours. *J. Natl Cancer Inst.*, **64,** 1307.

MISHELL, B. & SHIIGI, S.M. (1980). *Selected Methods in Cellular Immunology.* p. 131. W.H. Freeman and Co., San Francisco.

PELLIGRINO, M.A., FERRONE, S. & THEOFILOPOULOS, A.W. (1976). Isolation of human T and B lymphocytes with 2-amino-ethylisothiouronium bromide (AET) treated sheep red blood cells. *J. Immunol. Methods,* **11,** 273.

PREHN, R.T. (1977). Immunostimulation of the lymphodependent phase of neoplastic growth. *J. Natl Cancer Inst.*, **59,** 1043.

SAIKI, O. & RALPH, P. (1982). Induction of Human Immunoglobulin Secretion. *Cell. Immunol.*, **70,** 301.

SALMON, S.E., & BUICK, R.N. (1979). Preparation of permanent slides of intact soft agar colony cultures of haematopoietic and tumor stem cells. *Cancer Res.*, **39,** 1133.

SCHULTZ, R.M., CHIRIGOS, M.A. & OLKOWSKI, Z.L. (1980). Stimulation and inhibition of neoplastic cell growth by tumor-promoter-treated macrophages. *Cell Immunol.*, **54,** 98.

SIEGEL, F.P. & SIEGEL, M. (1977). Enhancement by irradiated T cells of human plasma cell production: dissection of helper and suppression function. *J. Immunol.*, **118,** 642.

SMALL, M. & TRAININ, M. (1976). Separation of populations of sensitized lymphoid cells into fractions inhibiting and fractions enhancing syngeneic tumor growth *in vivo. J. Immunol.*, **117,** 292.

TOROK-STORB, B., MARTIN, P.J. & HANSEN, J.A. (1981). Regulation of *in vitro* erythroporesis by normal T cells: Evidence for two T-cell subsets with opposing function. *Blood,* **58,** 171.

TOROK-STORB, B. & HANSEN, J.A. (1982). Modulation of *in vitro* BFU-e growth by normal Ia positive T cells is restricted by HLA-Dr. *Nature,* **298,** 473.

WILLIAMS, N., JACKSON, H., RALPH, P. & NAKOINZ, I. (1981). Cell interactions influencing murine marrow megakaryocytes. Nature of the potentiating cell in the bone marrow. *Blood,* **57,** 157.

WILLIAMS, W., BEUTLER, E., ERSLEV, A. & RUNDLES, R.W. (1977). *Hematology,* p. 1627, McGraw-Hill, New York.

YRON, P., WOOD, T.A., SPRESS, P. & ROSENBERG, S. (1980). *In vitro* growth of murine T cells. V. Isolation and growth of lymphoid cells infiltrating syngeneic solid tumors. *J. Immunol.*, **125,** 238.